# Yoga for hypertensive patients: a study on barriers and facilitators of its implementation in primary care

**DOI:** 10.1080/16549716.2021.1952753

**Published:** 2021-07-29

**Authors:** Raja Ram Dhungana, Shiva Ram Khatiwoda, Yadav Gurung, Željko Pedišić, Maximilian de Courten

**Affiliations:** a Institute for Health and Sport, Victoria University, Melbourne, Australia; b Patanjali Ayurveda Medical College and Research Center, Dhulikhel, Kathmandu, Nepal; c Child and Youth Health Research Center, Auckland University of Technology, Auckland, New Zealand

**Keywords:** Implementation, hypertension, nepal, primary health care, yoga

## Abstract

**Background:**

International guidelines for hypertension treatment recommend the use of yoga, particularly among low-risk patients. However, evidence is lacking on the implementation potential of health-worker-led yoga interventions in low-resource, primary care settings.

**Objective:**

To assess barriers to and facilitators of the implementation of a yoga intervention for hypertensive patients in primary care in Nepal.

**Methods:**

The study was conducted using focus group discussions, in-depth interviews, key informant interviews, and telephone interviews. Data were collected from the ‘Yoga and Hypertension’ (YoH) trial participants, YoH intervention implementers, and officials from the Ministry of Health and Population in Nepal.

**Results:**

Most YoH trial participants stated that: (1) it was easy to learn yoga during a five-day training period and practise it for three months at home; (2) practising yoga improved their health; and (3) group yoga sessions in a community centre would help them practise yoga more regularly. Most YoH intervention implementers stated that: (1) they were highly motivated to implement the intervention; (2) the cost of implementation was acceptable; (3) they did not need additional staff to effectively implement the intervention; (4) providing remuneration to the staff involved in the intervention would increase their motivation; and (5) the yoga programme was ‘simple and easy to follow’ and ‘easily performed by participants of any age’. The government officials stated that: (1) yoga is considered as a key health promotional activity in Nepal; and (2) the integration of the yoga intervention into the existing health care programme would not be too challenging, because the existing personnel and other resources can be utilised.

**Conclusion:**

While there is a good potential that a yoga intervention can be implemented in primary care, capacity development for health workers and the involvement of community yoga centres in the delivery of the interventions may be required to facilitate this implementation.

## Background

Yoga is a lifestyle therapy that is commonly used globally to improve physical and mental health. It encompasses different forms of practice, whereby the most widely used one commonly includes postures, breathing, and meditation exercises [1]. Results of a recent meta-analysis indicate that yoga is a promising antihypertensive lifestyle therapy that reduces systolic blood pressure and diastolic blood pressure in hypertensive individuals on average by 10 mmHg and 6 mmHg, respectively [2]. The blood pressure reduction seems to be greater among those who practise all three components of yoga combined more than three times per week as compared with those who practise only one component of yoga [2]. Similarly, the health benefit is greater following 12 weeks of yoga practice as compared with a shorter intervention period [3].

Most of the evidence on yoga and hypertension has emerged from a few countries. Wu et al. (2) found that, out of 56 yoga interventions for blood pressure reduction conducted between 1983 and 2018, 42.9% (*n* = 24) were from India, 16.1% (*n* = 9) were from South Korea, and 41.1% (*n* = 23) were from non-Asian countries such as Australia, the UK, and the US. A few studies are also available from China, Cuba, Jamaica, Japan, Sweden, and Thailand [3]. More studies from other countries are needed on the topic. This recommendation is in accordance with the United Nations declaration [4] that emphasises the importance of improving national capacities for dealing with non-communicable disease in low- and middle-income countries, such as Nepal. Findings on the effectiveness and implementation potential of yoga interventions in the primary care setting in Nepal may partially be generalised to other low- and lower-middle income countries with similar low-resource, primary care settings.

Many of the previous trials were conducted in healthy or normotensive populations [5–14], recruited participants from the workplace [9,11,15] or academic settings [8,10], and used the respective centres [8–11,14,16] or separate yoga studios [17–19] for the intervention. However, little evidence is available to demonstrate the impact of health-worker-led yoga interventions on blood pressure reduction in low-resource, primary care settings.

An intervention tested under ideal clinical trial conditions may fail to show the same effect(s) when implemented in real-world settings due to the dilution of effects and barriers associated with the implementation of the intervention [20]. The quality of the implementations of the interventions in real-world clinical settings is likely to be affected by various factors related to the implementation process, external environments, internal settings related to the implementing organisation, the stakeholders’ perspectives towards the intervention, policy priorities, and the level of acceptability of the intervention [21,22]. For example, a high acceptability of the intervention may positively affect the adherence to the treatment and, therefore, improve the outcomes of the intervention [23]. Likewise, high costs associated with an intervention may impede its implementation as well as its sustained delivery [24,25]. Therefore, having a good understanding of the factors that can influence the implementation of an intervention may improve the interpretation of its effectiveness [26]. It may be helpful when informing the relevant stakeholders and maximising the effectiveness when scaling up the intervention.

**Figure 1.**
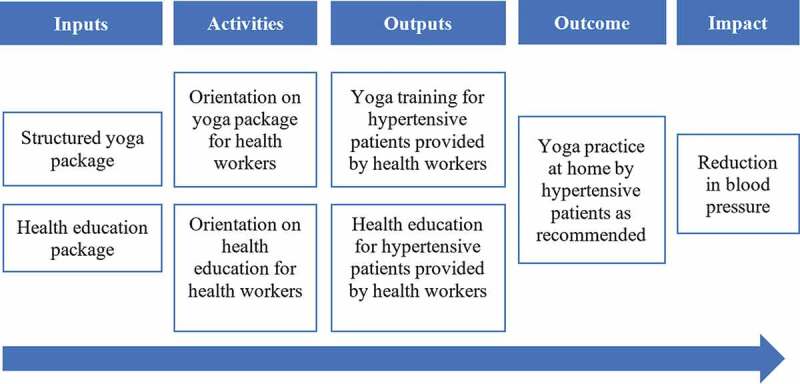
Logical framework of YoH trial

Between 2017 and 2018, a clinical trial entitled ‘Impact of a structured yoga program on blood pressure reduction among hypertensive patients’ (YoH) was conducted in seven Ayurveda Health Centres in Nepal [27]. This trial was carried out to test whether a health-worker-led yoga intervention implemented in the clinical practice setting could effectively reduce blood pressure in hypertensive patients. One hundred and twenty-one participants were randomised to the intervention (*n* = 61) and control (*n* = 60) groups. The intervention group received an intervention consisting of an initial five-day structured yoga training at the centres, followed by home-based practice of yoga (yoga postures for nine minutes, breathing exercise for nine minutes, and meditation and relaxation for twelve minutes) for five days a week over a period of 90 days (Figure 1). The study found that, after the intervention, the mean differences in systolic blood pressure and diastolic blood pressure between the intervention group and the control group were −7.66 mmHg and −3.86 mmHg, respectively [28].

The aim of this study was to investigate barriers to and facilitators of the implementation of the yoga intervention for blood pressure reduction among hypertensive patients in the primary care setting in Nepal.

## Methods

### Study design and theoretical framework

**Figure 2.**
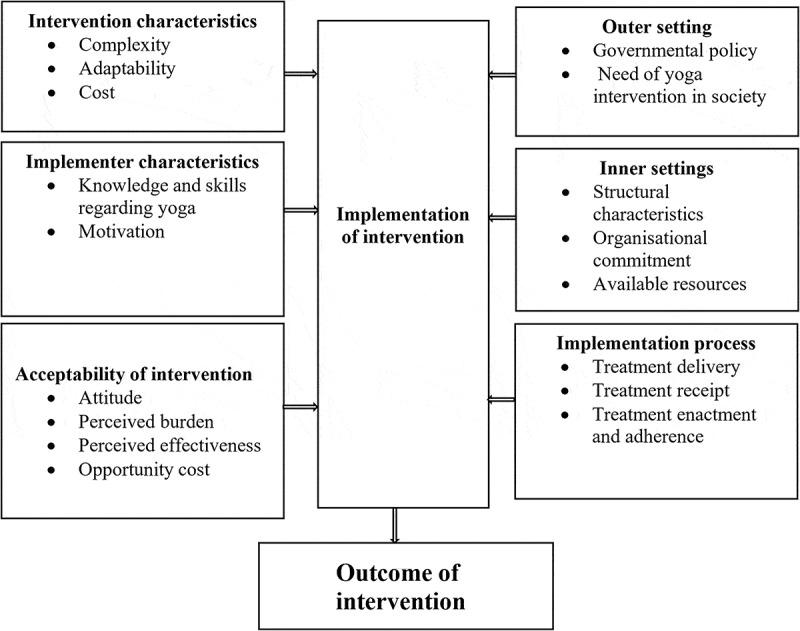
The elements of the Consolidated Framework for Implementation Research and the Theoretical Framework of Acceptability covered in this study

From 2019 to 2020, we conducted focus group discussions, in-depth interviews, key informant interviews, and telephone interviews to collect qualitative and quantitative data from the YoH trial intervention group participants, YoH intervention implementers, and officials from the Ministry of Health and Population in Nepal. To report the qualitative component of the study, we applied the COnsolidated criteria for REporting Qualitative research (COREQ) checklist [29] (Supplementary file 1). We used the Consolidated Framework for Implementation Research (CFIR) [21] and the Theoretical Framework of Acceptability (TFA) [22] to guide the collection, analysis, and interpretation of the qualitative data. The basic structure of the CFIR encompasses an interactive interplay between the intervention contents, context, and process of implementation. To gain a more comprehensive insight into the factors influencing implementation, the CFIR model was supplemented using TFA constructs (Figure 2). The TFA consists of seven constructs, including affective attitude, burden, ethicality, intervention coherence, opportunity costs, perceived effectiveness, and self-efficacy.

### Study setting

This study used data from the seven Ayurveda Health Centres where the original YoH trial was conducted. For the purpose of the current study, we collected additional data from six of the health centres located in Dhading, Kaski, Nuwakot, Ramechap, Rolpa, and Surkhet. These centres provide various preventive, promotive, and curative primary care services for people. The school yoga health programme, for example, is one programme that is delivered as a routine health promotion activity.

### Study participants

Data were collected from key stakeholders in the YoH trial, including the intervention group trial participants and the trial implementers (i.e. medical officers). To identify the government’s position on the use of yoga-related programmes in primary care settings, we also invited key officials from the Ministry of Health and Population and the Department of Ayurveda and Alternative Medicine to participate in the study.

### Data collection

**Figure 3.**
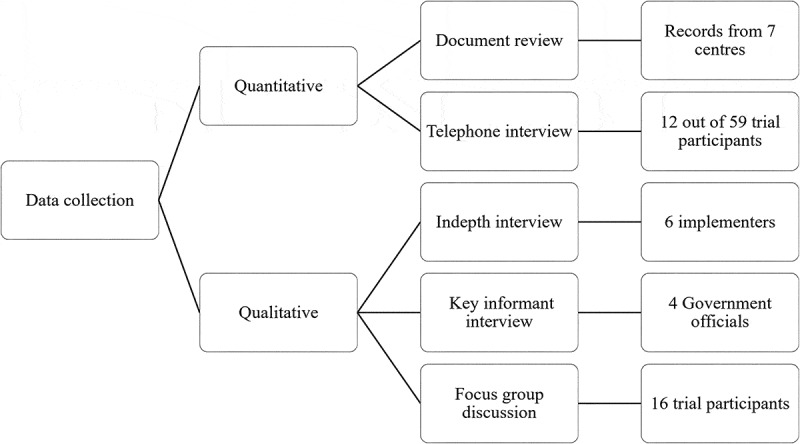
Data collection methods

We collected qualitative data by carrying out focus group discussions, in-depth interviews, and key informant interviews, while quantitative data were collected by telephone interviews and by reviewing the YoH trial documentation (Figure 3). The data collection tools were initially developed in English by one author (RRD) and reviewed independently by two other authors (MdC and ZP). The data collection tools were then translated into Nepali by a certified translator. We conducted a pilot focus group discussion and an interview to pre-test the tool. The information collected during the pilot test was used to revise the tool prior to the study.

#### Focus group discussions

Three focus group discussions were carried out, one in each of three randomly selected health centres in which the YoH trial was implemented. A total of 16 participants (six from the first trial centre, six from the second trial centre, and four from the third trial centre), who were originally in the intervention group of the YoH trial, were involved in the focus group discussions. We used a semi-structured interview guide to collect information about the participants’ perspectives and experiences regarding the yoga intervention.

#### In-depth interviews

In-depth interviews were conducted to explore the implementers’ perspectives and experiences regarding the implementation of the YoH trial. The implementers were health workers who recruited and monitored the trial participants and delivered the intervention. We also invited six medical officers who implemented the intervention in their trial centre to the interview.

#### Key informant interviews

We conducted four key informant interviews with leading officials from the Ministry of Health and Population. We asked them about: a) the government policy on implementing yoga for health promotion; and b) the likelihood that a yoga intervention can be scaled-up in the primary health care.

#### Telephone interviews

We conducted telephone interviews with twelve randomly selected individuals who were members of the intervention group in the YoH trial. The participants in the subsample were of similar age as the participants in the YoH study intervention group. However, the distributions of gender, caste, and occupation categories differed between the samples (Supplementary file 2). We used a structured questionnaire to gain an insight into yoga skills that participants acquired during the training sessions (treatment receipt) [30] and how they applied these skills while practising yoga at home (treatment enactment) [30].

#### Examination of records from the YoH trial

Data on the inner context of health centres and characteristics of the people who implemented the intervention were also extracted from the records of the YoH trial.

### Data management and analysis

Focus group discussions and interviews were conducted in a closed room, without any potential distractions from bystanders, at the study sites. Two trained researchers (RRD and SK) were involved in interviewing, recording, note-keeping, and transcribing the interview records. The transcripts were translated into English and imported into NVivo 12 (QSR International Pty Ltd, Melbourne, Australia) for further analysis. We used the five domains of CFIR and five domains of TFA as themes for the template analysis [31]. One of the two researchers (RRD) then generated the first coding template by thoroughly reading and considering each statement in the two transcripts; one was from focus group discussion and the other, from in-depth interview. Detailed information about the themes and codes is available in Supplementary file 3. Two other researchers (ZP and MdC) independently reviewed the codes and revised the first template. Field notes and analytic memos were used to interpret the findings. At the end of the process, as a means of validation, two authors (SK and YG) independently rechecked the final template and the underlying codes.

In addition, one author developed an analytic-summary cross table that contained contextual issues and included seven thematic areas in its rows and two intervention components in its columns. The factors were determined and labelled as barriers or facilitators based on their potential negative or positive influence on the implementation of the intervention. Quality checks were performed independently by an external reviewer who, for this purpose, reviewed the codes and coding results.

The quantitative data were presented as absolute frequencies and percentages of the participant responses across different categories. The analysis of quantitative data was performed using the IBM Statistical Package for the Social Sciences (SPSS) software, version 27 (SPSS Inc., an IBM Company, Chicago, IL, USA).

## Results

### Participant characteristics

Of the 16 focus group discussion participants, the majority were females (Table 1). Half of the participants from the focus group discussion had either attended primary school or not had any formal education and were homemakers, whereas all of the in-depth interview participants and key informants had a postgraduate degree and were government employees.

**Table 1.** Participant characteristics

Characteristics		FGD	IDI	KII	Telephone interview
		*n* (%)	*n* (%)	*n* (%)	*n* (%)
Age	30–40 years	3 (18.8)	5 (83.3)	1 (25.0)	3 (25.0)
	40–50 years	6 (37.5)	1 (16.7)	3 (75.0)	3 (25.0)
	50 years and older	7 (43.7)	0 (0)	0 (0.0)	6 (50.0)
Sex	Female	10 (62.5)	3 (50.0)	1 (25.0)	8 (66.7)
	Male	6 (37.5)	3 (50.0)	3 (75.0)	4 (33.3)
Education	No formal education	5 (31.3)	0 (0)	0 (0)	7 (58.3)
	Primary	3 (18.8)	0 (0)	0 (0)	0 (0)
	Secondary	6 (37.5)	0 (0)	0 (0)	3 (25. 1)
	Bachelor	1 (6. 2)	0 (0)	0 (0)	1 (8.3)
	Post-graduate	1 (6.2)	6 (100.0)	4 (100.0)	1 (8.3)
Occupation	Employed	7 (43.7)	6 (100.0)	4 (100.0)	5 (41.7)
	Homemaker	8 (50.0)	0 (0)	0 (0)	7 (58.3)
	Retired	1 (6.3)	0 (0)	0 (0)	0 (0)

FGD, focus group discussion; IDI, in-depth interview; KII, key informant interview

### General findings from the qualitative component of the study

**Figure 4.**
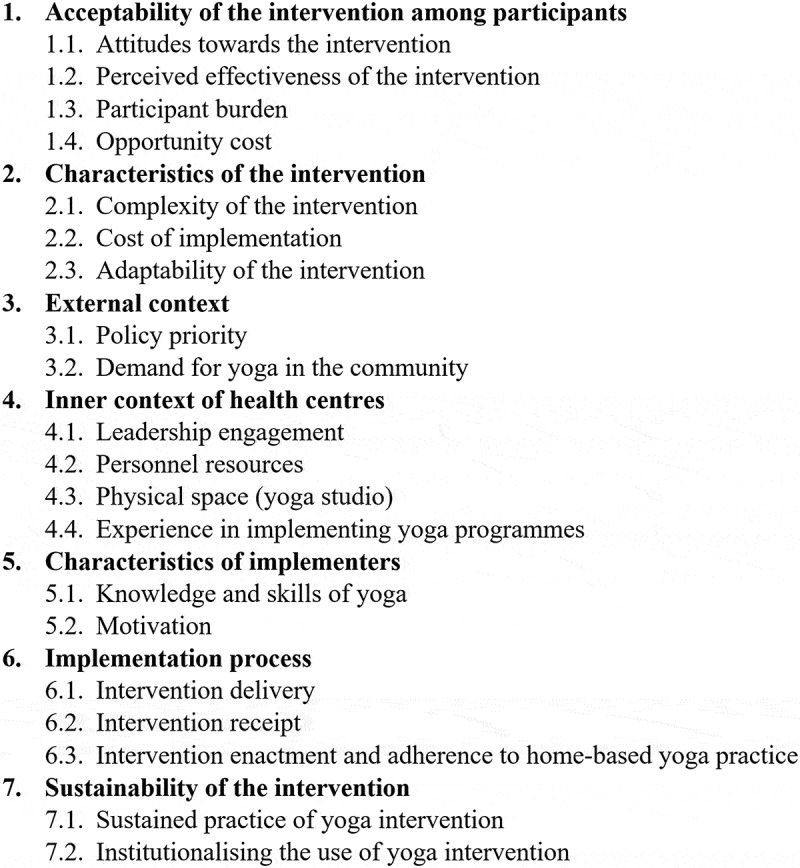
Factors influencing the implementation of yoga intervention in the primary care setting

Factors that are likely to influence the implementation of the yoga intervention were grouped into seven principal themes, namely, the (1) acceptability of the intervention among participants, (2) characteristics of the intervention, (3) external context, (4) inner context of health centres, (5) characteristics of implementers, (6) implementation process, and (7) sustainability of the intervention (Figure 4).

The findings across the seven thematic areas are summarized in Table 2 as barriers (*n* = 5) to or facilitators (*n* = 21) of the implementation of the yoga intervention.

**Table 2.** Hierarchy of evidence of promising research practices

Barriers and facilitators	Intervention component	
	Five-day, health-centre-based yoga training	Home-based yoga practice
*Attitude towards yoga intervention*		
Positive attitude towards intervention	**+**	**+**
*Burden*		
Short duration of a single yoga session (30 minutes)	**+**	**+**
Weekly frequency of home-based yoga sessions and overall duration of the intervention (5 days a week for 90 days)		-
*Opportunity cost*		
Competing priorities (e.g. household work)		-
*Perceived effectiveness*		
Belief that yoga has a positive health impact	**+**	**+**
Prioritising ‘instant relief’ types of treatment	-	-
*Intervention characteristics*		
Brevity and simplicity of the yoga programme	**+**	**+**
Low cost of implementation	**+**	**+**
Adaptability in regard to the mode of intervention delivery and time schedule (i.e. participants are free to practise yoga at home at any time of the day)	**+**	**+**
*External context*		
High priority of yoga in the government policy	**+**	**+**
High demand in the community	**+**	**+**
Low awareness of yoga as a health-promotion activity in rural communities	-	-
*Health-centre-related*		
Active participation of the health centre leadership in the implementation of the intervention	**+**	**+**
Existing personnel resources	**+**	**+**
Other available resources	**+**	**+**
Previous experience in implementing yoga programmes	**+**	**+**
Dedicated staff for yoga	**+**	**+**
High priority given to yoga	**+**	**+**
*Implementer characteristics*		
Good knowledge	**+**	**+**
High skills	**+**	**+**
High motivation (among lead implementers)	**+**	**+**
Low motivation (among supporting staff) due to a lack of financial incentive	-	-
*Implementation process*		
Delivery of the intervention according to its protocol	**+**	**+**
Ability of participant to perform yoga	**+**	**+**
*Sustainability of the intervention*		
Sustained practice of yoga	**+**	**+**
Institutionalising the use of yoga intervention	**+**	**+**

‘+’ sign denotes a facilitator; ‘-’ sign denotes a barrier

### Acceptability of trial

#### Attitude towards yoga intervention

The intervention participants showed a positive attitude towards the yoga intervention in general, yoga training they received in the health care centres, yoga instructors, and yoga home-based practice. They were happy to be involved in the intervention. They considered yoga to be a healthy lifestyle intervention and considered it as highly important in their daily routines. They believed that by practising yoga, one could help prevent various chronic diseases. Most participants were also satisfied with the training they received from the trial centre. They preferred group-based training, as they thought practising yoga in a group is more interactive and fun than practising alone. They liked practising relaxing or meditating postures the most. They said that practising *Savasana* [Corpse] and *Yoganidra* [Yogic sleep] made them feel happy and relaxed. The majority of them also believed that 30 minutes was the optimal duration of yoga sessions for them. Few participants mentioned that they would rather have had shorter practice sessions.

#### Perceived effectiveness of the intervention

Almost all intervention participants said that they experienced improvements in their health after doing yoga. The commonly perceived improvements were: *blood pressure reduction; feeling calm, light, fresh, relaxed, and well; having a good appetite; anger reduction; and reduced pain, stiffness, and discomfort in muscles and joints*. The participants who had a positive attitude towards the intervention reported more benefits than others.

#### Participant burden

Some yoga intervention participants perceived attending yoga training at the health centre for five days as a time-consuming and burdensome task. They also reported that engaging in the same yoga programme every day was tiring. Intervention participants also mentioned that the schedule for home-based yoga practice overlapped with their household and business activities and that they sometimes needed to appoint someone to take over their duties while they practised yoga.

#### Opportunity cost

None of the intervention participants explicitly mentioned opportunity costs incurred in the YoH trial. However, some of the YoH trial implementers thought that three months of follow-up might have been too long for them. They said it was difficult to track patients for three months and expressed concerns about the costs associated with the additional staff time needed to monitor the home-based yoga practice over such a long period.

### Intervention characteristics

#### Complexity of the intervention

When responding to the questions about the complexity of the intervention, most YoH trial implementers stated that the yoga programme was ‘*well-structured’* (in-depth interview participant 1 [IDI-1]), ‘*simple and easy to follow*’ (IDI-5), ‘*without too many and too complicated postures*’ (IDI-2), and ‘*easily performed by participants of any age*’ (IDI-3). The intervention participants generally considered the intervention package to be ‘*easy*’ to practise, and they felt ‘*comfortable*’ while doing it.

#### Adaptability of the intervention

Some implementers suggested that the yoga programme should be made more adaptable, as they noticed that some of the young intervention participants requested more strenuous yoga activities than the relaxing ones. The implementers said they could not fulfil the request, because they followed the strict intervention protocol.

Some implementers also reported that the trial participants who lived near the trial centres wished to engage in the yoga training under their supervision at the health centre for longer than just five days. The implementers could not fulfil the request, because they had to follow the intervention protocol.

#### Cost of implementation

Implementers of the intervention said that the cost of implementation was acceptable. They said there were ‘*no major expenses’* (IDI-1) other than the ‘*cost of printing [protocol, training manual] and papers’* (IDI-2), which was easily covered from the ‘*internal/organisational funds*’ (IDI-3). They mentioned that all supporting staff agreed to work voluntarily, which otherwise would have been challenging to manage from the available funds. However, most of the implementers mentioned that paying the supporting staff in the intervention by the hour motivated them even more strongly to implement the intervention (more details about this provided in the section entitled ‘Characteristics of Implementers/Motivation’).

### External environment and context

#### Policy priority

The government officials unanimously agreed that yoga is considered to be a key health promotional activity in Nepal and that several yoga-related programmes have been implemented in Ayurveda Health Centres. They also stated that, alongside the Ministry of Health and Population, other ministries such as the Ministry of Education and the Provincial Government had also assigned due priority to yoga and supported the inclusion of yoga-related activities in the school health programmes.

A few implementers thought otherwise and argued that the local government was not assigning due importance to yoga programmes.

‘They [government] do not give as much priority [to yoga] as they give to other programmes like immunization and reproductive health programmes.’ (IDI-2)

#### Demand for yoga intervention in the community

The intervention participants generally thought that a high demand for yoga programmes existed among the hypertensive individuals in the community. They said that some of their hypertensive friends and acquaintances had expressed interest in taking part in the intervention. Most of the government official and implementers of the intervention also agreed that more and more people in the community seem to be interested in doing yoga. Some intervention participants also suggested that yoga interventions should be offered as part of the routine health care, so that the wider hypertensive community can reap the benefits they gained during the intervention.

An implementer of intervention from Rolpa, a relatively remote, rural place in Nepal, however, had a different opinion. This implementer thought that the people from Rolpa were not clearly aware of the benefits of yoga and said that these people wanted instant relief for their ailments instead.

A government officer expressed the same opinion by stating that people in remote areas have a relatively low health literacy and need to prioritise maintaining the security of their livelihoods.

‘Usually, yoga is advised to be practised in the morning. In rural areas, women often take care of family and livestock. They often cook [for the family], cut hay for cattle, and collect firewood and water.’ (KII-1)

One focus group participant from a rural area also expressed their concern regarding the competing priorities and said:

‘I had to do a lot of household work. I had to look after children. I sometimes could not follow the routine of [yoga] practice at home.’

### Inner contexts and setting of trial centres

#### Leadership engagement

All implementers of the intervention who were also in charge of their health centres stated that they were actively involved in planning and implementing the intervention (Table 3). They also provided yoga training to the intervention participants, mobilised the supporting staff to coordinate recruitment, organised the training sessions, and monitored the participants.

**Table 3.** Characteristics of health centres that implemented the yoga intervention

Centre	Leadership engagement^†^	Personnel resources (Instructors) ^‡^	Yoga studio^§^	Experience in implementing yoga programme^||^
Dhading	Yes	health assistant and medical officer	Yes	Yes
Kaski	Yes	medical officer	Yes	Yes
Nuwakot	Yes	health assistant	No	Yes
Ramechap	Yes	medical officer	Yes	Yes
Rolpa	Yes	medical officer	No	Yes
Rupandehi	Yes	health assistant	Yes	Yes
Surkhet	Yes	health assistant and medical officer	Yes	Yes

^†^Involvement of the medical officer in planning and execution of the trial

^‡^Dedicated personnel resources for instructing yoga

^§^Whether the yoga training studio is inside the trial centre

^||^Experience in implementing yoga programmes

#### Personnel resources

The health centres had at least seven staff each, including a medical officer, *Kaviraj* [Ayurvedic health assistant], *Vaidya* [auxiliary health assistant], and other staff. Of these staff, the medical officer and health assistants had knowledge and skills in yoga and were involved in providing yoga training to the intervention participants. Most of the intervention implementers felt that they did not need additional staff to effectively implement the intervention. For example, IDI-1 said:

‘We don’t need separate staff. Available staff can handle that [yoga intervention].’

#### Physical space (yoga studio)

Five centres had their own yoga studio where they could provide training to the participants. The health centre located in Rolpa did not have a yoga studio, but the participants used the outpatient department room to organise the yoga training in the YoH trial. The intervention implementers from Nuwakot stated that they organised the yoga training outside the health centre. The yoga studios in the remaining five health centres are well-equipped.

#### Experience in implementing yoga programmes

The implementers of the intervention and government officials noted that many District Ayurveda Health Centres, including the seven trial centres, had been implementing different yoga-related programmes like *Swostha Jivan Karyakram* [Healthy Life Programme] and *Vidyalaya Yog Shiskya Karyakram* [School Health Programme]. They also reported that yoga interventions have a relatively high priority as compared to other, similar programmes.

### Implementers’ characteristics

#### Yoga knowledge and skills among health workers

All intervention implementers were trained in yoga and had a medical degree (Table 4). Some of them were volunteering as yoga instructors in the community outside of their office hours. They stated that the supporting health care workers, such as *Kaviraj* [Ayurvedic health assistant] and *Vaidya* [auxiliary health assistant], who were involved in implementing the yoga intervention, also had previous knowledge and skills in yoga (Table 3).

**Table 4.** Implementers’ characteristics

**Participants**	**Yoga knowledge**		**Yoga skill**		**Motivation for implementation**	
Health centre	Lead implementer^†^	Other staff^‡^	Lead implementer	Other staff^‡^	Lead implementer	Other staff^‡^
Dhading	Yes	Yes	Yes	Yes	Yes	NS^§^
Kaski	Yes	Yes	Yes	Yes	Yes	Yes
Nuwakot	Yes	Yes	Yes	NS	Yes	NS
Ramechap	Yes	Yes	Yes	NS	Yes	NS
Rolpa	Yes	Yes	Yes	Yes	Yes	Yes
Rupandehi	Yes	Yes	Yes	Yes		
Surkhet	Yes	Yes	Yes	NS	Yes	Yes

^†^Lead implementer of YoH trial and participant in the current study

^‡^Supporting staff

^§^Not sufficient

However, some of the intervention implementers questioned whether the supporting health workers would be competent enough as yoga instructors, unless they would receive adequate professional training.

‘Our assistants are not fully competent in yoga … If we give them good orientation and train them for 7-8 days, they can implement the yoga programme very well.’ (IDI-5)

One of the implementers said that yoga instructors need a good knowledge of yoga as well as a ‘*flexible*’ body, which would only be possible if they regularly practised yoga. They added that the health workers lacked practical experience in yoga instruction. Accordingly, two government officials suggested providing yoga training to health care workers before scaling up the yoga intervention in other centres.

#### Motivation

The implementers of the intervention were highly motivated to contribute to the delivery of the intervention. They believed that Ayurveda Health Centres and their employees should offer yoga programmes for patients. However, some of the intervention implementers stated that their supporting staff were less motivated to implement the intervention. They suggested that providing remuneration or other incentives to the staff involved in the intervention would increase their motivation.

### Implementation process

#### Intervention delivery

All intervention implementers stated that they followed the protocol and provided five days of training to the participants, as planned. The training was provided one-on-one or in a group session. All the centres provided yoga training to the participants in the morning (before their office work). Although this was not specified in the intervention protocol, they thought it would be best for the patients to learn yoga in the morning.

#### Intervention receipt

The intervention participants were satisfied with the five-day yoga training. Most of them reported that they could understand and perform yoga as instructed during the training. The intervention implementers were also confident that the intervention participants were interested in attending the five-day yoga training and that they were able to gain sufficient yoga skills during the training.

#### Intervention enactment and adherence to home-based yoga practice

Most of the intervention participants found it easy to practise yoga during the three-month, home-based part of the intervention. Most of them also reported that they regularly practised yoga during the intervention. Some participants stated they could not practise yoga regularly, although they were motivated to do so. The most frequently cited challenges to practising yoga at home were time constraints and competing priorities (e.g. looking after children and grandchildren). Participants sometimes skipped their yoga sessions or shortened the sessions by only practising *Pranayam* [breathing exercise], if they were in a rush.

The intervention implementers also agreed that some participants may not have strictly followed the intervention protocol. They mentioned that the reasons for this could be a lack of commitment to the yoga practice, the low perceived effectiveness of the yoga intervention, a lack of family support, and competing, work-related priorities.

Most of the participants suggested that offering group yoga sessions in a community centre would help them practise yoga more regularly. Those who worried that they would disturb their household members if they practised yoga early in the morning and those who felt distracted by their children were particularly in favour of the idea of group sessions in a community-based yoga centre.

### Sustainability of the intervention

#### Sustained practice of yoga

A year after the completion of the yoga intervention trial, the majority of the intervention participants reported that they had continued practising yoga. Some of the participants stated that they are motivated to continue practising yoga over a long term. A few participants quit practising yoga after the study period due to time constraints.

#### Institutionalising the use of yoga intervention

One intervention implementer stated that, after the YoH trial, his health centre incorporated the yoga intervention into their routine practice. They mentioned that the centre now recommends all eligible hypertensive patients to practise yoga. The government officials and intervention implementers suggested that the integration of the yoga intervention into the existing health care programme would not be too challenging. Moreover, the yoga intervention would not require additional resources, if it could utilise the existing personnel and other resources.

### Quantitative findings

More than two-thirds of the participants found the training useful for them, considered that it helped them to learn yoga, and were satisfied with the training they received. The most commonly reported barriers to regular home-based yoga practice were the burden of repeating the same routine over and over again (cited by 16.7% of participants), comorbid conditions (cited by 16.7% of participants), and other priorities at home (cited by 16.7% of participants; Table 5).

**Table 5.** Results of telephone interviews with intervention participants

Question	*n* (%)
Did you attend yoga training?	
Yes	12 (100)
How useful was the training for you to learn yoga?	
Very useful	7 (58.3)
Useful	2 (16.7)
Somewhat useful	3 (25.0)
Not useful	0 (0)
Not at all useful	0 (0)
How satisfied were you with the yoga training?	
Very satisfied	9 (75.0)
Satisfied	3 (25.0)
Somewhat satisfied	0 (0)
Not satisfied	0 (0)
Not at all satisfied	0 (0)
Did you practise yoga at home regularly as instructed?	
Yes	12 (100)
No	0 (0)
Did you find any barriers in home-based yoga practice?	
Yes	6 (50.0)
No	6 (50.0)
What were the barriers? (*n* = 6)	
The routine is difficult to follow	2 (333)
I don’t have necessary skills	0 (0)
I have other medical conditions	2 (33.3)
I feel too lazy	1 (16.7)
I am not convinced about its positive effects	0 (0)
I have family/work commitments	2 (33.3)
It is too physically demanding	0 (0)
I lack motivation	1 (16.7)
I lack time	1 (16.7)
It is too inconvenient	0 (0)
It is boring	1 (16.7)
I am too tired	0 (0)

## Discussion

The main finding of this study is that a simple, health-worker-led yoga intervention for blood pressure reduction among hypertensive patients has a good potential for implementation in the primary care setting. Specifically, we found that implementers, trial participants, and government officials perceived yoga as an effective, simple, low-cost, and scalable intervention for hypertension control.

Our results show that study participants had a positive attitude towards the intervention, a positive perception of the effectiveness of yoga for blood pressure reduction, and a relatively low participant burden. This indicates that the yoga intervention was highly acceptable. The positive attitudes that the participants expressed toward the intervention could be explained by the fact that yoga has a long tradition in Nepal [32]. Yoga originated and spread across South Asia, including Nepal, around 2000 years ago [1,33]. It is now a culturally accepted and widely practised lifestyle therapy in Nepal. Given its popularity, yoga has recently been included in the secondary school curriculum [32,34,35]. Because yoga is a very popular activity in many other countries in South Asia, such as India [36], similarly positive attitudes of individuals towards yoga interventions could be expected across South Asia. It should be noted that yoga is also one of the most common exercise forms and recreational activities in a number of other countries. For example, in 2016, around 10% of the US population practised yoga [37]; therefore, it can be assumed that a positive attitude towards yoga interventions is likely to be held in the primary care settings in a range of other countries as well. This assumption, however, needs to be confirmed in future studies.

The stakeholders’ perceptions of the complexity and relative advantages and disadvantages of an intervention may influence the effectiveness of its implementation [38]. The interventions that are being perceived as less complex are more likely to be implemented effectively [38]. In our study, the stakeholders perceived the intervention package as ‘*easy to follow*’ and ‘*not complicated*’, which is promising for its future implementation. Furthermore, the implementation cost is negatively associated with the effectiveness of implementation and sustained delivery of an intervention [24,25]. We found that the cost associated with the implementation of yoga intervention was negligible, as the health centres largely relied upon the existing personnel and other resources. Although the low complexity and cost of implementation of the yoga intervention suggest that it has a good potential for implementation in the primary care setting in Nepal, our findings indicate that the adaptability of the intervention package should be improved to meet varying patient requirements. In addition, health centres should incentivise the supporting staff to increase their motivation to adequately deliver the intervention.

This study confirmed some of the previously identified external and intra-organisational factors that are likely to facilitate the effective implementation of a health-promoting intervention [39]. Mendel et al. described six sets of factors that indicate the ability of an organisation to implement and maintain the delivery of a new intervention [40]. Organizational structure, resource availability, and policies are some examples. In the current study, we found that the government policy assigns high priority to the use of yoga for health promotion, that a high demand exists for yoga among hypertensive patients, and that Ayurveda Health Centres have adequate personnel and other resources required for the implementation of a yoga intervention. This suggests that implementation readiness for a yoga intervention is present at all levels in Nepal, from the government to individual health centres. Nevertheless, it is essential to improve the knowledge and skills of health workers in yoga instruction.

Our study also shed light on barriers associated with home-based yoga practices among hypertensive patients. The most commonly cited barriers were a lack of a daily routine, time constraints, and competing priorities (particularly household work and occupational commitments), findings that are in line with those of a previous study [41]. The use of educational videos to guide home-based yoga practice family support and replacing home-based yoga practice with group-based yoga practice in community centres are two commonly recommended possible strategies to overcome the challenges of home-based yoga practice.

The findings of our study may have implications regarding the inclusion of yoga interventions in the routine practices at health centres. An improved understanding of the factors influencing the effectiveness of implementation of the yoga intervention may contribute to scaling-up the YoH and similar interventions in other primary health-care centres in Nepal and potentially other countries. Nepal already has national policy and mechanisms that promote yoga as a lifestyle therapy. For example, the Multisectoral Action Plan for Prevention and Control of NCDs (2014–2020) and the Urban Health Policy (2015) envision yoga as a health promotional tool for NCD prevention and control. Similarly, there are several yoga-based health promotional interventions, such as the *Swatha Jiwan karyakram* [English: Healthy Life Program] and *Vidhaylaya yoga shiskya karyakram* [School Yoga Education Program], that are supported by the Ministry of Health. The current study findings may help policy makers extend the existing yoga-based community interventions to the primary care setting for the management of hypertension and potentially other NCDs. Our findings may also be used to encourage policy makers in other countries to consider designing policies that would promote the use of yoga interventions in the primary care setting.

The limitations of the study are due to its retrospective design, which does not allow the study findings to be used to address concerns encountered during the implementation of YoH trial. The findings may, however, be used to improve the future implementation of the YoH intervention and similar health-promotion initiatives. The retrospective design of the study may also have resulted in a recall bias among the participants. If possible, future interventions should consider combining prospective, real-time, and retrospective feedback from participants, health workers, and other stakeholders.

## Conclusion

The majority of the evaluated factors, such as the acceptability of the intervention, external environment, organisational attributes, and characteristics of implementers, indicate that there is a good potential for the implementation of the yoga intervention in a low-resource, primary care setting. The wider implementation of yoga interventions may require the capacity development of health workers and involvement of community yoga centres in the delivery of the interventions. These findings have greater implications in Nepal and other countries that have similar settings in terms of understanding how the factors influence the implementation of yoga in primary care as a lifestyle therapy. However, as the contextual factors may vary across different countries, the study findings may not necessarily be generalisable outside Nepal. Future studies on this topic in other countries are needed to confirm our findings.

## Supplementary Material

Supplemental Material

## Data Availability

The study materials are available from the corresponding author upon a reasonable request.

## Paper context

Yoga is rarely used for hypertension treatment in primary care. The reasons for this have not been sufficiently explored. This study investigated barriers to and facilitators of the implementation of a yoga intervention for hypertensive patients in the primary care setting in Nepal. The findings may inform stakeholders about the implementation potential of yoga interventions for hypertension control. The findings are generalisable to Nepal and potentially to other countries with low-resource primary care settings.
