# Supplementary material for: Yoga for hypertensive patients: a study on barriers and facilitators of its implementation in primary care
Source: Glob Health Action. 2021 Jul 29;14(1):1952753. doi: 10.1080/16549716.2021.1952753 (PMC8330799; doi:10.1080/16549716.2021.1952753)
Supplement: Supplemental Material [file ZGHA_A_1952753_SM0341.zip › Supplementray files/Supplementary file 2. Characteristics of subsample .docx]

| **Table 1. Characteristics of the YoH intervention group and its subsample used in the current study** | | | |
| --- | --- | --- | --- |
|  |  | **Total (*n=*59)** | **Subsample (*n*=12)** |
| Characteristics |  | % | % |
| Age in years; mean (SD) |  | 47.1 (1.4) | 49.5 (3.8) |
| Gender | Male | 42.6 | 33.3 |
|  | Female | 57.4 | 66.7 |
| Caste | Brahman | 27.9 | 41.7 |
|  | Chhetri | 21.3 | 16.7 |
|  | Janajati | 41.0 | 25.0 |
|  | Others | 9.8 | 16.7 |
| Occupation | Employed | 57.4 | 41.7 |
|  | Homemakers | 39.3 | 58.3 |
|  | Others | 3.3 | 0.0 |
