# Supplementary material for: Yoga for hypertensive patients: a study on barriers and facilitators of its implementation in primary care
Source: Glob Health Action. 2021 Jul 29;14(1):1952753. doi: 10.1080/16549716.2021.1952753 (PMC8330799; doi:10.1080/16549716.2021.1952753)
Supplement: Supplemental Material [file ZGHA_A_1952753_SM0341.zip › Supplementray files/Supplementary file 2. Template of prior themes and codes.docx]

**Table 1. Template for themes and codes with definitions used in the qualitative analysis**

| **Name of themes and codes** | **Definition** |
| --- | --- |
| **1.  Acceptability of the intervention among participants** | |
| 1.1.   Attitudes towards the intervention | Attitudes of the intervention participants towards yoga training, yoga instructors, and home-based practice |
| 1.2.   Perceived effectiveness of the intervention | Whether the participants experienced any changes in their health as a result of the intervention |
| 1.3.   Participant burden | Burden to intervention participants caused by the intervention |
| 1.4.   Opportunity cost | The loss of potential gain while participating in the yoga intervention |
| **2.  Characteristics of the intervention** |  |
| 2.1.   Complexity of the intervention | Implementers' perspectives regarding the complexity of the intervention |
| 2.2.   Cost of implementation | Costs associated with the implementation of the intervention |
| 2.3.   Adaptability of the intervention | To what extent was the intervention adaptable to different circumstances and situations |
| **3.   External context** |  |
| 3.1.   Policy priority | Government's policy regarding yoga in Nepal |
| 3.2.   Demand for yoga in the community | The demand for a yoga intervention among hypertensive people |
| **4.  Inner context of health centres** |  |
| 4.1.   Leadership engagement | Whether any officials from the health centres were involved in implementing the intervention or not |
| 4.2.   Personnel resources | Personnel resources for implementing a yoga intervention, particularly the availability of yoga instructors |
| 4.3.   Physical space (yoga studio) | Whether yoga studios are available in the health centres |
| 4.4.   Experience in implementing yoga programme | Previous experience of the health centres in implementing yoga-related programmes |
| **5.    Characteristics of implementers** |  |
| 5.1.   Knowledge and skills of yoga | Implementers' knowledge and skill, particularly regarding the academic or vocational training in yoga instruction |
| 5.2.   Motivation | The motivation of the intervention implementers for participating in the delivery of the intervention |
| **6.  Implementation process** |  |
| 6.1.   Intervention delivery | Whether the 5-day yoga training was delivered as intended |
| 6.2.   Intervention receipt | Whether the participants received or learned yoga intervention as planned |
| 6.3.   Intervention enactment and adherence to home-based yoga practice | Whether the participant could perform home-based yoga intervention regularly or not |
| **7.  Sustainability of the intervention** |  |
| 7.1.   Sustained practice of yoga intervention | Whether participants kept practising yoga even after the completion of the intervention |
| 7.2.   Institutionalising the use of yoga intervention | Health centre integrating yoga intervention in routine care |
